# Overview of Risk Factors and Prevention of Capsular Contracture Following Implant-Based Breast Reconstruction and Cosmetic Surgery: A Systematic Review

**DOI:** 10.7759/cureus.10341

**Published:** 2020-09-09

**Authors:** Enkhmaa Luvsannyam, Dhara Patel, Zaira Hassan, Swetha Nukala, Manoj R Somagutta, Pousette Hamid

**Affiliations:** 1 Department of Research, California Institute of Behavioral Neurosciences & Psychology, Fairfield, USA; 2 Neurology, California Institute of Behavioral Neurosciences & Psychology, Fairfield, USA

**Keywords:** capsular contracture, breast implant, breast reconstruction

## Abstract

Capsular contracture is one of the most common complications of implant-based breast reconstruction or augmentation surgery. Despite advanced molecular biology, the exact mechanism of this complication is not fully understood. PubMed was searched for studies, published from 2015 to 2020, focused on potential risk factors and preventions of capsular contracture (CC) in patients who underwent implant-based breast surgery. A total of 533 articles were identified from PubMed, and 13 articles were selected ultimately for our review after eligibility screening and quality appraisal. Common risk factors of CC include biofilm, surgical site infections (SSI), history of prior CC or fibrosis, history of radiation therapy, and implant characteristics. Interventions that decrease the rate of CC include antibiotic prophylaxis or irrigation, acellular dermal matrix (ADM), leukotriene (LTE) inhibitors, surgical techniques, and others. Multiple risk factors are proposed to be a component of the pathophysiology of CC. However, there is inconsistent evidence supporting these risk factors, and the current data was based on broad heterogeneous studies. While efforts are being undertaken to solve this complication with improved technologies and surgical practices, CC remains to be unsolved. Our objective was to provide a summary of the current data of contributing risk factors as well as preventative and treatment measures for CC.

## Introduction and background

Breast cancer is the most frequently diagnosed cancer in women around the world, and up to 41% of patients who undergo mastectomy receive breast reconstruction [1]. Breast implant has been present since the 1960s, and 65% of reconstruction surgery is implant-based in the United States [2]. The main goals of breast reconstruction are to reshape the breast due to tissue loss following breast cancer; to revise and fix the previous reconstruction surgery; and to augment the breast for cosmetic purposes. Along with its advantages for physical and psychological satisfaction given for the patients, complication rates are high following implant-based breast reconstruction especially for capsular contracture (CC).

CC is a distressing complication of breast implant surgery and often requires revision operation. Up to half of the patients develop CC, and 30% of them suffer from CC with Baker rates III and IV following implant-based breast reconstruction [3,4]. Risk factors found to be associated with CC include previous capsular fibrosis, radiation therapy, contamination with biofilm-producing bacteria, surgical site infections (SSI), and immune response to the foreign material [3,5]. The expression of toll-like receptor 4 is also seen in peri-implant tissue fibrosis and may play a role in myofibroblast differentiation to induce CC development [6]. The exact mechanism of the pathophysiology of CC formation is still unknown. An infection has been linked with the formation of CC extensively. Generally, breast surgery is considered to be a clean surgery but the postoperative SSI rate rises by 2%-2.9% in augmentation and is the most common cause of readmission [7,8]. The common organisms identified are *Staphylococcus epidermidis* and *S. aureus*, Escherichia, Pseudomonas, Propionibacterium, and Corynebacterium [9,10].

While the procedures for breast surgery and pre- or postoperative interventions are being improved, the causes and prevention methods for CC remain unclear. This study aims to review the risk factors associated with CC and to outline the available preventative and treatment measures to reduce the rate of CC.

## Review

Methods

Protocol

The protocol of this systematic review follows the Preferred Reporting Items for Systematic Reviews and Meta-Analyses Protocol (PRISMA-P) 2009 guideline.

Search Strategy, Study Selection, and Data Extraction

Electronic databases PubMed Central (PMC) and Medical Subject Heading (MeSH) were searched for articles. The keywords included in the search strategy include: "Implant Capsular Contracture/microbiology" OR "Implant Capsular Contracture/prevention and control" OR "Implant Capsular Contracture/statistics and numerical data"; "Mastectomy/complications" OR "Mastectomy/mortality" OR "Mastectomy/psychology"; AND "Surgical Wound Infection/analysis" OR "Surgical Wound Infection/microbiology" OR "Surgical Wound Infection/mortality" OR "Surgical Wound Infection/prevention and control" OR "Surgical Wound Infection/statistics and numerical data". No language restrictions were applied.

Three authors screened the titles and abstracts using the inclusion and exclusion criteria to identify the eligibility of the studies. Reference duplicates were manually checked by one author. The inclusion criteria were studies within the last five years (2015-2020) and studies with only human subjects that focused on CC and SSIs following breast surgery. We included various types of studies except for letters to the editors, animal and in vitro studies. Two authors extracted the following data from each study independently: study title, publication date, study design, sample size, mean follow-up, and mean age of the patients.

**Figure 1:**
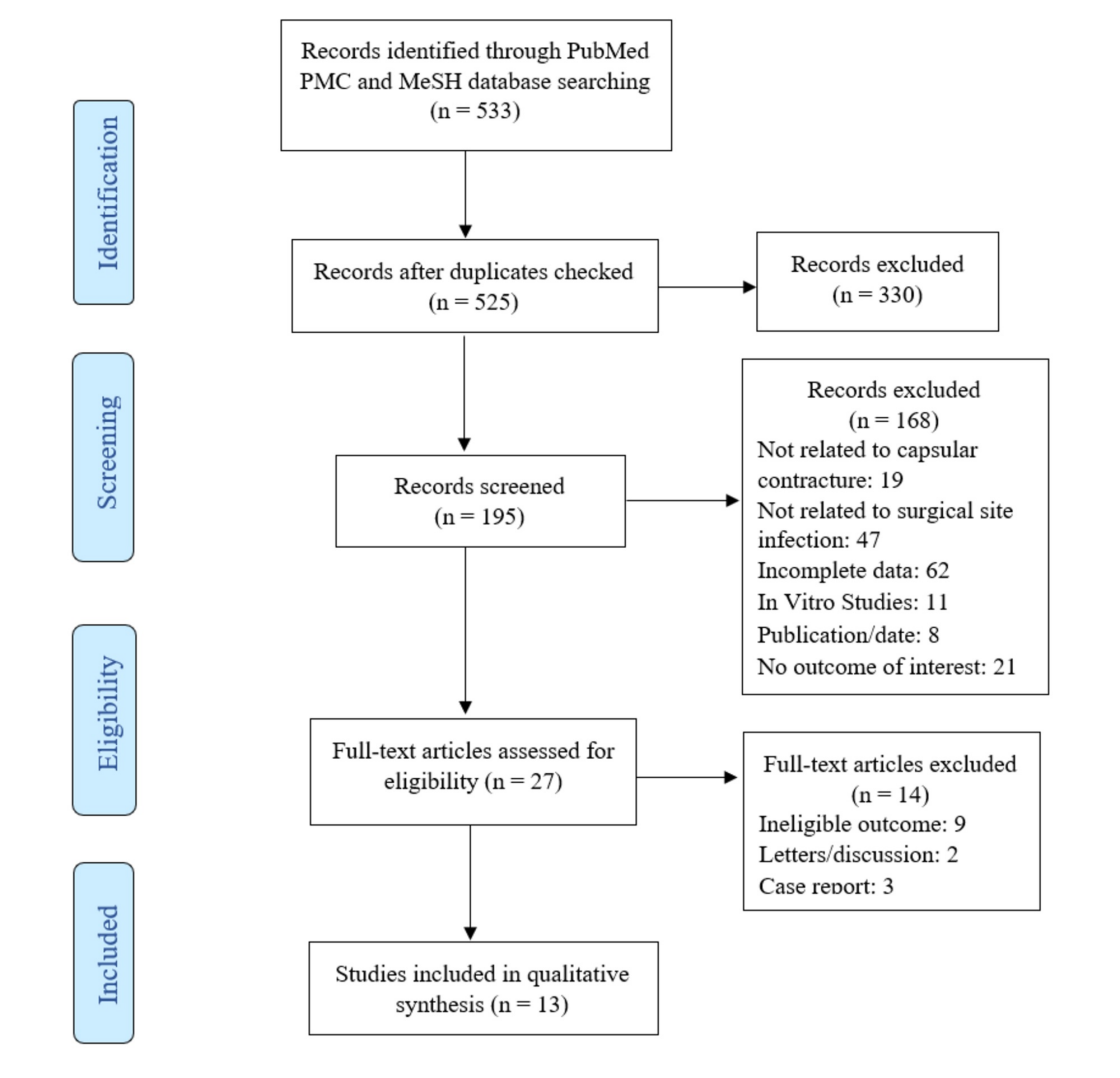
The PRISMA diagram showing identification, screening, and eligibility of the literature retrieved

We identified 533 publications total from PubMed PMC and MeSH search and excluded 520 publications due to duplication, ineligibility, incomplete data, and irrelevance to our topic. Thirteen studies were ultimately selected and included in our review. Preferred Reporting Items for Systematic Reviews and Meta-Analyses (PRISMA) flowchart diagram of literature retrieval is shown in Figure 1.

Quality of the Studies and Risk of Bias Within Studies

Four authors assessed the quality of included studies and risk of bias using the Newcastle-Ottawa Scale (NOS) for observational studies; A Measurement Tool to Assess Systematic Reviews (AMSTAR 2) for systematic reviews and meta-analysis; and Cochrane risk of bias tool for randomized controlled trials (RCTs).

Results

Study Characteristics

The 13 selected articles include two RCTs, one controlled trial (CT) without randomization, two cohort studies, one systematic review, one meta-analysis, one cross-sectional study, three retrospective studies, and two traditional reviews. Patients in the included studies are women who underwent either mastectomy followed by implant-based reconstruction surgery or implant-based breast augmentation only. The study characteristics are summarized in Table 1.

**Table 1:** Characteristics of the Included Studies (n = 13)

Authors, Year [References]	Study Design	Location	Sample Size	Mean Follow-Up (months)	Mean Patient Age ± SD (years)
Tanner, 2018 [4]	Retrospective analysis	England	214	20.2	40 ± 12.6
Ajdic et al., 2016 [5]	Traditional review	United States	N/A	N/A	N/A
Palubicka et al., 2019 [9]	Retrospective analysis	Poland	2129	N/A	55 ± 13.1 (range: 34.1-74.6)
Sinha et al., 2017 [11]	Prospective cohort study	United States	1024	12	48.4 ± 10.6
Samargandi et al., 2018 [22]	Systematic review	United States	N/A	N/A	34.3 (5 studies) Not specified (2 studies)
Viola et al., 2019 [23]	Randomized controlled trial	United States	241	6	53 (range: 32-76, infected patients) 47 (range: 22-81, uninfected patients)
Sobti et al., 2020 [29]	Cohort study	United States	47	25.3 ± 10.8 (prepectoral group) 27.0 ± 11.3 (subpectoral)	50.8 ± 11.3
Bresnick, 2017 [38]	Nonrandomized controlled trial	United States	1122	6	Range: 22-60
Poppler et al., 2015 [40]	Retrospective cross-sectional study	United States	26	N/A	49.6 ± 5.5 (range: 40-64)
Hai et al., 2020 [41]	Meta-analysis	United States	N/A	N/A	N/A
Straub et al., 2015 [42]	Traditional review	United States	N/A	N/A	N/A
McCarthy et al., 2012 [43]	Randomized controlled trial	United States	70	12	51 (range: 29-72)
Papadopoulos et al., 2018 [44]	Retrospective study	Germany	15	11.9 (range: 3-30)	49.2 (range: 22-72)

Overall Results and Recommendations of the Studies

Multiple risk factors are mentioned to be associated with CC across studies. The number of studies discussed each topic include 14 studies about infection with biofilm-producing bacteria and SSIs; six studies about radiation therapy and previous fibrosis; nine studies about antibiotics and irrigations; six studies about implant surface, size, and placement; three studies about acellular dermal matrix (ADM); three studies about leukotriene antagonists (LTE); one study about toll-like receptor 4; and one study about Thy1(CD90) expression. The suggested risk factors and preventions for CC are summarized in Table 2.

**Table 2:** Summary of the Risk Factors Associated With Breast Implant Capsular Contracture ALCL, anaplastic large-cell lymphoma; TLR, toll-like receptor; TGF, transforming growth factor; SSI, surgical site infection; CHG, chlorhexidine gluconate; CC, capsular contracture.

Risk Factors	Prevention	Treatment	Additional Comment/Recommendation
Implant surface	Textured implants instead of smooth implants; antibiotics for biofilm-producing bacteria.	Capsulectomy for definitive treatment; open capsulotomy	Microtextured implants show lower rates of CC compared to macrotextured implants. Textured implants may contribute to a higher risk of biofilm productions compared to the smooth implant. Future study indication: association between textured implants, biofilm, and ALCL.
Toll-like receptor 4	Targeting toll-like receptor 4 expressed by fibroblasts and myofibroblasts	Reducing the expression of TLR 4 and its correlation with estrogen receptor-β	Future indication: more evidence-based trials in the association between TLR 4 and capsular fibrosis, as well as its correlation with estrogen receptor-β expression.
Biofilm SSI	Preoperative antiseptic skin agents; Antibiotic and saline pocket irrigation; antibiotic mesh; nipple shield (prevention from endogenous breast flora); surgical techniques	IV antibiotics; capsulectomy for definitive treatment	Lower rate of biofilm-related CC seen in CHG as preoperative antiseptic, inframammary incision approach, and subpectoral implant placement. There is no difference between a triple antibiotic and saline irrigation in the incidence of biofilm-related CC.
Radiation therapy	Radiation dose adjustment; limitations of tissue volume and duration of therapy	Primarily symptomatic treatment; inhibition of TGF-β and associated signaling molecules; capsulectomy or open capsulotomy	Radiation increases postoperative complication rates regardless of timing. Prepectoral breast reconstruction has a lower rate of CC in an irradiated patient vs. subpectoral breast reconstruction. Future study indication: molecular mechanisms of fibrosis to reduce the inflammatory responses and control myofibroblast development.
Previous fibrosis			Previous fibrosis formation significantly increases the risk of future CC development.
Thy1(CD90) expression	Targeting Thy1 expression in fibroblasts; salinomycin (decreased Thy1 expression)	Reduction of Thy1 expression to prevent further development of fibrosis	Depletion of Thy1 prevented myofibroblast formation in capsular fibroblasts and significantly decreased α-smooth muscle actin and collagen levels. Future study indication: anti-scarring ability of salinomycin in radiation-induced CC.

SSI

SSI is a major postoperative complication that can cause CC formation. The risk of SSI is higher with reconstruction performed during the primary surgery, and the incidence rate of acute infection is higher than that of late infections [7,8,9]. A recent study states that SSIs are underestimated in implant-based breast reconstructions because the majority of them occur later than 30 days following surgery, causing persistent inflammation and subsequent formation of late-onset CC [11]. Implant removal after breast reconstruction surgery has shown to be associated with hypertension, elevated BMI > 25, and diabetes, but the infection is the most common cause of implant loss [12-15]. Post breast implant surgery morbidity has an association with operation time. Longer operation time has significantly increased the length of hospital stay due to complications such as infection [16]. Moreover, postoperative drains aid in the prevention of seroma and bacterial infection; however, prolonged drain use has significantly increased the rate of SSI, and early drain removal is encouraged as early as postoperative day seven [17,18].

Antibiotics and Irrigations

Preoperative skin antiseptic agents are known to reduce postoperative complications. CC rate is reduced with povidone-iodine and antimicrobial irrigations [19]; however, chlorhexidine gluconate was found to be more effective than povidone-iodine for reduction of biofilm-related CC [20]. A study has found that there is no difference between triple antibiotic vs. saline irrigation in the reduction of CC incidence [21]. The available evidence suggesting antimicrobial irrigation in the reduction of CC is weak and inconclusive [22].

Staphylococci species are the most common axillary flora, and antibiotics targeted at these species do not show a significant impact on SSIs [23]. Preoperative prophylaxis has not significantly reduced SSIs in breast cancer surgery [24], and prolonged postoperative antibiotic prophylaxis also has not shown to decrease implant loss or highly virulent infections [25]. Patient compliance plays an important role in preventing SSIs, and medication noncompliance doubles the risk of infection in breast surgery [26]. In primary breast augmentation, most organisms in acute infections are Gram-positives and are adequately covered by a single dose of IV cephalosporin; clindamycin or vancomycin is recommended in individuals with β-lactam allergies [8]. The antibiotic is broadened with fluoroquinolones or vancomycin in late infections or secondary surgeries due to mixed organisms with both Gram-positives and Gram-negatives [8].

Radiation Therapy and Implant Surface

Post-mastectomy radiation therapy leads to higher rates of CC [27]. Several studies report that patients who had radiation therapy are more likely to experience reconstruction failure due to complications. The expression of Thy1 (CD90), which has an important role in scar tissue formation, is shown to be increased by radiation; thus, targeting the Thy1 receptor may decrease the rate of radiation-induced fibroproliferation of capsular tissue [28]. Muscle fibrosis is another possible contributor to CC in irradiated patients with subpectoral implant placement vs. prepectoral implant placement [29].

Breast implant characteristics especially implant surface seem to play a role in CC. Studies have analyzed that smooth implants, compared to textured implants, are significantly associated with CC, and the choice of the textured implant may reduce the risk of CC [4,30]. Moreover, microtextured implants may have lower rates of CC compared to macrotextured implants [4]. However, macrotextured implants have been associated with increased risk of anaplastic large-cell lymphoma (ALCL) significantly compared to smooth or microtextured implants [31,32]. Breast implant-associated ALCL is a rare complication and may have an infectious cause as seen by the bacterial biofilm on the implant [33].

ADM and Leukotriene (LTE) Antagonists

Long-term study has shown that the incidence of CC remains low in patients who had implant-based breast reconstruction with ADM with or without radiation therapy and states that it may truly prevent early-onset CC [34]. Due to the early formation of peri-implant fibrosis, ADM may play an important role in preventing the CC formation rather than delaying the formation [34]. In terms of the difference between the matrices, fenestrated acellular matrices are not significantly different than nonfenestrated matrices in the reduction of CC rates [35]; however, meshed acellular matrices significantly decrease the rates of minor complications including postoperative drain duration, narcotic use, and length of stay vs. unmeshed matrices [36].

LTE antagonists have been known to prevent and treat CC. Multiple studies have found that the patients who used LTE antagonists, either montelukast or zafirlukast, have significantly decreased rate of CC compared to the control group [37-39]. Although there is a short-term benefit in CC reduction rates with the use of LTE antagonists, its long-term side effects such as liver damage are not known in depth [38].

Discussion

CC remains the most frequently recorded complication and cause of reoperation following breast implant surgery; yet, the pathophysiology of CC has not been clearly defined, and the prevention remains to be unresolved [30]. Multiple literature reviews analyzed the risk factors associated with CC and preventative measures, and the findings are inconclusive and lack evidence. The purpose of this systematic review was to analyze the risk factors, etiology, and preventions for CC and to provide recommendations according to the current literature.

Risk Factors of CC

Microbial biofilm is challenging to treat and can cause chronic inflammation and the formation of capsular fibrosis [3,5]. While most studies support that infection with biofilm-producing bacteria leads to the development of CC, few studies found no correlation among biofilm and CC. A study suggests that confirming the presence of biofilm is difficult and requires direct visualization of the colony [40]. Moreover, bacteria may form biofilm for its self-defense from unfavorable surroundings, and biofilm may not be the cause of CC, instead, it is the environmental trigger causing both biofilm and fibrosis independently [40].

SSIs are a common cause of breast reconstruction failure following breast surgery [17]. The association between SSI and CC is unclear and lacks evidence. Patients undergoing a mastectomy followed by immediate implant-based reconstruction surgery have twice the rate of SSI than patients without immediate reconstruction surgery [7]. The appropriate antibiotics are recommended less than 60 minutes before the incision to be more effective [8]. Although antibiotics are widely used to prevent SSI, the antimicrobial prophylaxis in breast surgery is controversial. Studies have found that pre- and postoperative antibiotics did not significantly decrease the rate of SSIs [23]; and peri-operative prophylaxis also has no impact in preventing SSI in breast cancer surgery [24]. Extensive use of antibiotics increases the risk of antibiotic resistance and disrupts the normal gastrointestinal flora resulting in Clostridium difficile-related pseudomembranous colitis [41]. There is not enough evidence supporting that antibiotic irrigation of the implant pocket plays a role in CC prevention [22].

Implant surface may or may not affect biofilm formation. The textured implants may cause higher rates of biofilm formation compared to smooth implants, but a study also found no difference between textured and smooth implants in biofilm formation [5]. A recent study suggests that microtextured implants contribute to a low rate of CC formation; however, this study did not have a control group, and a randomized study with different types of implant textures would be useful in understanding the association between implant surface and formation of CC [4]. Deeper research is also warranted to prove the relationship between macrotextured implants and ALCL due to a lack of evidence.

All the studies support that post-mastectomy radiation therapy is a strong risk factor for postoperative complications including CC and SSIs [9,29,34]. The formation of radiation-induced fibrosis is mainly affected by the radiation dose, the volume of tissue, and the duration of the therapy [42]. Patients with systemic lupus erythematosus, systemic scleroderma, Marfan syndrome, and specific genetic mutations are more prone to radiation-induced fibrosis [42]. Although there is no proven intervention to prevent radiation-induced CC, reduction of inflammation and matrix synthesis has a crucial role in the aims of therapeutic development. More specifically, targeting TGF-β and its associated signaling molecules is important in the management approach [42].

Management Approaches to CC

ADM is a popular intervention to reduce the rate of CC. An RCT has found that ADM does not reduce postoperative pain [43], but another study states that meshed ADM has significantly reduced overall complications including postoperative pain [36]. Multiple studies mention the promising outcomes of ADM in the incidence of CC; however, most of these studies were short of duration. Therefore, independent, long-term studies with controlled groups are indicated for use of ADM in the prevention of CC.

LTE antagonists are used by many plastic surgeons to some extent for the treatment of CC, but there is no clear evidence of the benefit of these medications currently. The available information on how LTE antagonists work on the pathogenesis of CC is limited. Several studies have shown favorable outcomes with LTE antagonists in decreasing the incidence of CC and support the use of these medications in a safe way [37-39]. However, these studies lack evidence on the prevalence and side effects of long-term use, highlighting the need for future studies to determine the clinical efficacy and safety as well as to develop a clear standard treatment protocol.

A recent study demonstrated fat grafting in women with the previous CC to restore the soft tissue thickness and found that it significantly reduces pain and tension due to CC [44]. The study suggests that lipofilling can reduce CC grade and reduce fibrotic damage, especially after radiation-induced CC [44]. Although the study showed a promising effect of lipofilling in CC treatment, the study had a small sample size and high risk of bias; therefore, further studies with larger sample size and control groups are warranted.

Currently, the gold standard treatment of CC is capsulectomy to remove the capsule and replace it with a new implant. Surgeons performed open capsulotomy, which does not remove the capsule from the body and reinserts the intact implant, more often in the past. A retrospective study suggests that an open capsulotomy is a safe treatment and there is no significant difference between capsulectomy and open capsulotomy in the CC recurrence rate [45]. However, this study consists of small sample size and does not provide adequate evidence. Open capsulotomy would also be not useful if the underlying etiology of CC is an infection.

Limitations

There are several limitations to our study. First, the studies included in this review were obtained from a single database and were mixed studies with heterogeneity in terms of sample size, follow-up duration, control groups, and randomization, which may create bias in reporting the development and different stages of CC. Second, we selected studies from 2015 to 2020, except one study in 2012 [45], to have the most current data; however, this may cause missing of the valuable information from previous studies. We were not able to obtain all the full articles relevant to our topic; thus information taken was limited to abstracts only. Third, only two studies were RCTs, and one study was CT without randomization; therefore, more RCTs are needed for stronger evidence.

Future Directions

Given the limited number of studies with evidence-based medicine on this topic, well-designed studies are indicated in the future. According to the current literature, the incidence of SSI and CC has decreased with antibiotic prophylaxis, textured implant, ADM, leukotriene antagonists, and an open capsulotomy; however, these interventions have not been proved. The important question to be addressed should be more focused on the pathogenesis of CC, which has been debatable. What exactly is the cause of CC? Is it multifactorial? What is the biggest factor contributing to the pathogenesis of CC among other factors? Focusing on these questions will help further studies to search for strategies about particular preventions and treatment approaches for CC.

## Conclusions

CC is most likely to be multifactorial, and the exact mechanism of pathogenesis of CC formation is unknown. The available evidence on risk factors associated with CC is weak and inconclusive. Our review suggests that infectious cause may be the strongest risk factor of CC etiology, and further studies on this aspect are required. The current literature data on prevention and treatment of CC is heterogeneous, and results are controversial. Greater efforts in developing modern imaging and technologies will continue to provide advanced tools to understand the pathophysiology of CC in depth and further develop preventative and treatment interventions.
